# Role of GRK6 in the Regulation of Platelet Activation through Selective G Protein-Coupled Receptor (GPCR) Desensitization

**DOI:** 10.3390/ijms21113932

**Published:** 2020-05-30

**Authors:** Preeti Kumari Chaudhary, Sanggu Kim, Youngheun Jee, Seung-Hun Lee, Kyung-Mee Park, Soochong Kim

**Affiliations:** 1 College of Veterinary Medicine, Chungbuk National University, Cheongju 28644, Korea; chaudharypreety11@gmail.com (P.K.C.); tkdrnfld@naver.com (S.K.); dvmshlee@cbu.ac.kr (S.-H.L.); parkkm@chungbuk.ac.kr (K.-M.P.); 2 College of Veterinary Medicine and Veterinary Medical Research Institute, Jeju National University, Jeju 63243, Korea; yhjee@jejunu.ac.kr

**Keywords:** GRK6, GPCR, PAR, ADP, desensitization, platelets

## Abstract

Platelet G protein-coupled receptors (GPCRs) regulate platelet function by mediating the response to various agonists, including adenosine diphosphate (ADP), thromboxane A_2_, and thrombin. Although GPCR kinases (GRKs) are considered to have the crucial roles in most GPCR functions, little is known regarding the regulation of GPCR signaling and mechanisms of GPCR desensitization by GRKs in platelets. In this study, we investigated the functional role of GRK6 and the molecular basis for regulation of specific GPCR desensitization by GRK6 in platelets. We used GRK6 knockout mice to evaluate the functional role of GRK6 in platelet activation. Platelet aggregation, dense- and α-granule secretion, and fibrinogen receptor activation induced by 2-MeSADP, U46619, thrombin, and AYPGKF were significantly potentiated in GRK6^−/−^ platelets compared to the wild-type (WT) platelets. However, collagen-related peptide (CRP)-induced platelet aggregation and secretion were not affected in GRK6^−/−^ platelets. Interestingly, platelet aggregation induced by co-stimulation of serotonin and epinephrine which activate G_q_-coupled 5HT_2A_ and G_z_-coupled α_2A_ adrenergic receptors, respectively, was not affected in GRK6^−/−^ platelets, suggesting that GRK6 was involved in specific GPCR regulation. In addition, platelet aggregation in response to the second challenge of ADP and AYPGKF was restored in GRK6^−/−^ platelets whereas re-stimulation of the agonist failed to induce aggregation in WT platelets, indicating that GRK6 contributed to P2Y_1_, P2Y_12_, and PAR4 receptor desensitization. Furthermore, 2-MeSADP-induced Akt phosphorylation and AYPGKF-induced Akt, extracellular signal-related kinase (ERK), and protein kinase Cδ (PKCδ) phosphorylation were significantly potentiated in GRK6^−/−^ platelets. Finally, GRK6^−/−^ mice exhibited an enhanced and stable thrombus formation after FeCl_3_ injury to the carotid artery and shorter tail bleeding times, indicating that GRK6^−/−^ mice were more susceptible to thrombosis and hemostasis. We conclude that GRK6 plays an important role in regulating platelet functional responses and thrombus formation through selective GPCR desensitization.

## 1. Introduction

Platelet aggregation is important to maintain hemostasis and thrombosis. Various agonists including thrombin, adenosine diphosphate (ADP), and thromboxane, mediate their cellular effects through the G protein-coupled receptors (GPCRs) to induce platelet activation, and these GPCRs have been the most common target for anti-thrombotic drug development. ADP-induced platelet aggregation requires co-activation of both the P2Y_1_ and P2Y_12_ receptors that couple to G_q_ and G_i_, respectively, and concomitant signaling from G_q_ and G_i_ is necessary for ADP-induced platelet aggregation [1]. Thromboxane A_2_ (TxA_2_) exerts its actions via the G protein-coupled thromboxane A_2_ receptor (TP receptor) and requires co-activation of G_q_ and G_i_ pathways to cause platelet aggregation [2,3,4]. However, it has been shown to activate G_i_ pathways through the P2Y_12_ receptor activation by the secreted ADP. Similarly, thrombin and thrombin receptor-activating peptides mediate their effect via the G protein-coupled protease-activated receptors (PARs), by direct coupling to G_q_ and G_12/13_. PARs also cause G_i_ stimulation indirectly through the P2Y_12_ receptor activation by the secreted ADP [5]. It has also been shown that co-stimulation of serotonin and epinephrine, which acts via the G_q_-coupled 5HT_2A_ receptor and the G_z_-coupled α_2A_ adrenergic receptor, respectively, induces platelet aggregation [6]. On the other hand, collagen and collagen-related peptide (CRP) activate platelets by stimulating tyrosine phosphorylation of the Fc receptor (FcR) γ-chain that contains an immuno-receptor tyrosine-based activation motif (ITAM) through the Ig receptor, glycoprotein (GP)VI [7].

The G-protein-coupled receptor kinases (GRKs) consist of seven different genes, of which two are exclusively expressed in rod and cone cells (GRK1 and 7), respectively. One has a very limited expression in the cerebellum, testis, and kidneys (GRK4), and the remaining four are expressed ubiquitously in mammalian tissues (GRK2, GRK3, GRK5, and GRK6) [8,9,10]. All GRKs share a similar basic structure with an N-terminal, a central catalytic domain, and a distinguishing C-terminal domain [11]. The N-terminus shares considerable homology, which has been considered to be responsible for receptor recognition, rendering it a critical structure for the selective targeting of GPCRs, while the C-terminal region consists of phosphorylation sites and key binding sites to PIP2 and Gβγ [12]. GRK6 is constitutively associated with the cell membrane through the N-terminal and C-terminal basic amino acid residue motifs. While there is some evidence of substrate specificity among the different members of the GRK family, most show activity towards a wide variety of agonist-occupied receptors in vitro.

The GRKs are known to have the ability to interact with the activated conformation of seven-transmembrane receptors besides heterotrimeric G proteins [13]. GRKs are recruited concurrently to agonist bound-activated GPCRs. Upon agonist stimulation, GRKs phosphorylate agonist-activated GPCRs on serine and/or threonine residues located in the C-terminal tail region and/or the third cytoplasm loop, leading to lower active coupling to the heterotrimeric G protein and the termination of the signaling, excluding further interaction between the GPCR and G protein [12]. This process is termed as receptor desensitization. It is used by the vast majority of GPCRs to turn off receptor-mediated signal transduction pathways [11,12,14]. GRK5 has been shown to play a crucial role in the mechanism of thrombin-induced desensitization of PAR1 in endothelial cells [15], and co-expression of GRK3 (but not GRK2) with PAR1 has been shown to inhibit thrombin-activated G_q_ signaling in *Xenopus* oocytes [16]. Desensitization of P2Y_12_ receptor in 1321N1 cells has been shown to be mediated by GRK2 and GRK6 [17]. It has been shown that GRK6 plays a major role in oxytocin receptor desensitization in the uterine smooth muscles [18]. GRK5 and GRK6 have been shown to regulate phosphorylation and desensitization of the TPα receptor [19], while TPβ receptor internalization is regulated by GRK2 in human embryonic kindey 293 (HEK293) cells [20]. In addition, it has been demonstrated that GRK2 and GRK3 are primarily responsible for agonist-dependent receptor phosphorylation and functional uncoupling, whereas GRK5 and GRK6 make lesser contributions to this outcome [21].

Although the contribution of GRK isoforms in the regulation of specific GPCR desensitization have been reported in other cells, little is known about the role of GRKs in GPCR-mediated desensitization in platelets. It is important that the responsiveness of platelets to various agonists be tightly regulated to avoid inappropriate thrombosis or excessive bleeding. Given the crucial role of agonists such as ADP, TxA_2_, and thrombin in platelet activation, characterization of the role of GRKs in GPCR-mediated platelet responses may reveal novel regulatory mechanisms regulating platelet function.

This study was therefore undertaken to evaluate the functional role of GRK6 and its molecular basis for regulation of GPCR desensitization in platelets using GRK6 knockout mice. We have shown that GRK6 selectively regulates specific GPCR-mediated platelet aggregation and secretion. We have further shown that GRK6 plays an important role in ADP and PAR4 receptor desensitization and regulates both G_q_- and G_i_-mediated signaling in platelets. Moreover, GRK6 contributes to thrombus formation in vivo. Therefore, we conclude that GRK6 is crucial for regulating platelet functional responses through selective GPCR desensitization.

## 2. Results

### 2.1. GRK6 Selectively Regulates GPCR-Mediated Platelet Functional Responses

**Figure 1:**
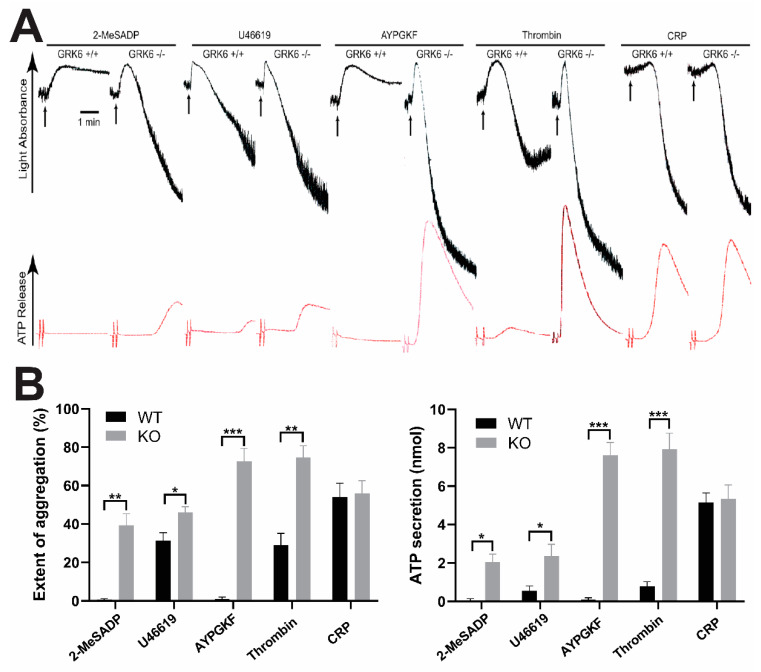
Agonist-induced platelet aggregation and dense granule secretion in the GPCR kinase (GRK)6-deficient platelets. Washed platelets from GRK6^−/−^ mice and GRK6^+/+^ littermates were stimulated with G-protein-coupled receptor (GPCR) agonists 30 nM 2MeSADP, 50 nM U46619, 60 µM AYPGKF, 0.1 unit/mL thrombin and GPVI agonist 2.5 µg/mL collagen-related peptide (CRP) for 3.5 min under stirring conditions. (**A**) Platelet aggregation (top) and ATP secretion (bottom) were measured in a lumi-aggregometer. All tracings shown are representative of at least three different experiments. (**B**) Quantification of extent of aggregation and dense granule secretion from panel A. Data are presented as mean ± SE *, *p* < 0.05; **, *p* < 0.01; ***, *p* < 0.005.

To determine the contribution of GRK6 to platelet function, we measured the various agonists-induced platelet aggregations and dense granule secretions in the wild-type (WT) and the GRK6-deficient mouse platelets. As shown in Figure 1, platelet aggregation and dense granule secretion induced by GPCR agonists including 2-MeSADP, U46619, AYPGKF, and thrombin were significantly potentiated in the GRK6-deficient platelets compared to those in the WT platelets. We found that the dose response curves were left-shifted, and there was little difference between WT and GRK6^−/−^ platelet in response to high dose of agonists. The extent of potentiation of the platelet function in response to U46619 was not as significant as it was for the other GPCR agonists. However, platelet aggregation and dense granule secretion in response to Glycoprotein VI (GPVI) agonist CRP were not affected in the GRK6-deficient platelets, indicating that GRK6 selectively regulated platelet aggregation and secretion in response to GPCR agonists.

**Figure 2:**
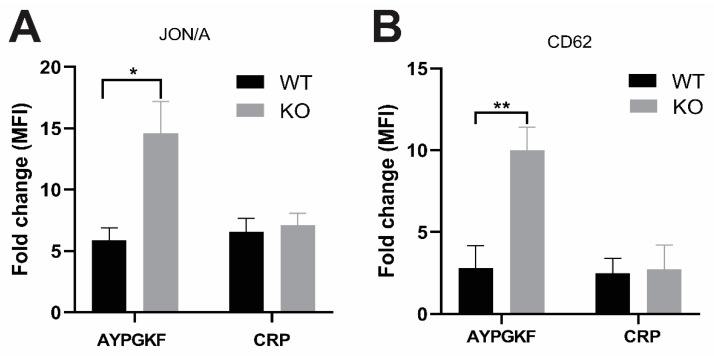
Integrin αIIbβ3 activation and α granule secretion in GRK6-deficient platelets. Washed platelets from GRK6^−/−^ (knockout, KO) mice and GRK6^+/+^ (wild-type, WT) littermates were stimulated with 100 µM AYPGKF and 2.5 µg/mL CRP in the presence of phycoerythrin (PE)-labeled JON/A antibody to determine integrin α_IIb_β_3_ activation (**A**) or FITC-labeled anti-P-selectin (CD62) antibody to evaluate α-granule secretion (**B**) by flow cytometry. Mean fluorescence intensities (MFI) are shown as fold change from unstimulated control. Data are representative of three independent experiments and are presented as mean ± SE. *, *p* < 0.05; **, *p* < 0.01.

A similar approach was adopted to analyze the activation of integrin αIIbβ3 by measuring the binding of the conformation-dependent antibody JON/A, and α-granule secretion by measuring the binding of an fluorescein isothiocyanate (FITC)-labeled anti-P-selectin antibody that recognizes the α-granule protein: P-selectin, by flow cytometry. Consistent with the results shown in Figure 1, we found that both JON/A binding and P-selectin expression induced by AYPGKF were significantly increased in GRK6-deficient platelets compared to WT platelets (Figure 2A,B). However, CRP-induced JON/A binding and P-selectin binding to GRK6^−/−^ platelets were not affected compared to WT platelets (Figure 2A,B). These results show that GRK6 plays an important role in the regulation of platelet functional responses downstream of GPCR pathways.

### 2.2. GRK6 Regulates Specific GPCR-Mediated Platelet Aggregation

**Figure 3:**
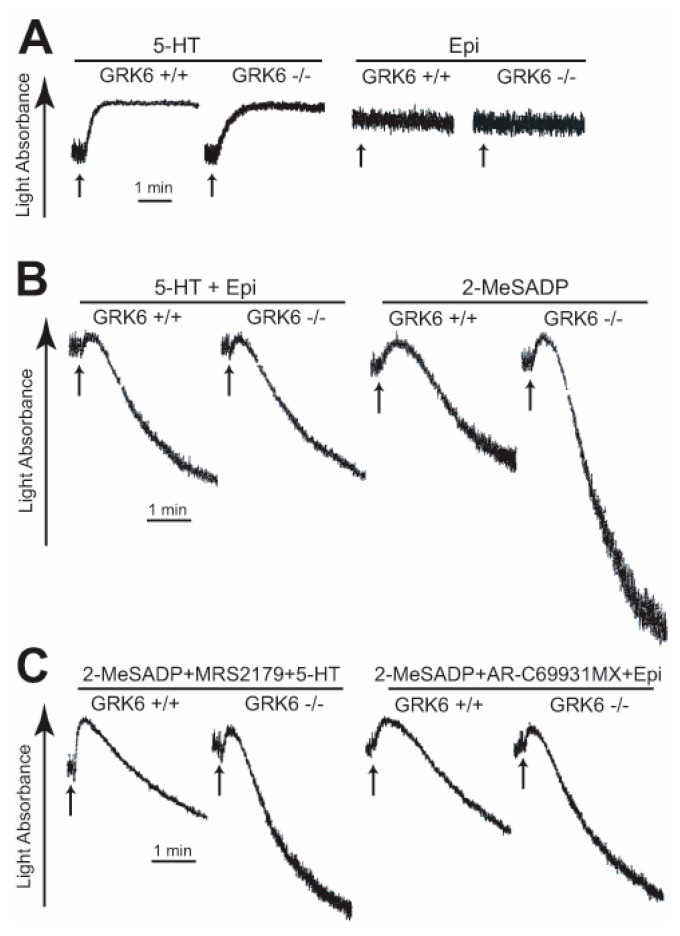
GRK6 selectively regulates P2Y_1_ and P2Y_12_ receptor-mediated platelet responses. Platelets from GRK6^+/+^ and GRK6^−/−^ mice were stimulated with (**A**) 20 µM 5-HT and 100 µM epinephrine, (**B**) 20 µM 5-HT + 100 µM epinephrine and 30 nM 2-MeSADP, and (**C**) 30 nM 2-MeSADP + 100 µM MRS2179 + 20 µM 5-HT and 30 nM 2-MeSADP + 100 nM AR-C69931MX + 100 µM epinephrine, and platelet aggregation was measured. Tracings are representative of three independent experiments.

It is known that ADP-induced platelet aggregation requires co-activation of the G_q_-coupled P2Y_1_ and the G_i_-coupled P2Y_12_ receptors while platelet aggregation induced by co-stimulation of serotonin (5HT) and epinephrine bypasses the P2Y_1_ and P2Y_12_ receptors to activate the G_q_-coupled 5HT_2A_ receptor and the G_z_-coupled α_2A_ adrenergic receptor, respectively [6]. In order to evaluate the role of GRK6 in the differential regulation of GPCRs, we measured the platelet aggregation induced by co-stimulation of serotonin and epinephrine in the WT and the GRK6-deficient platelets. Interestingly, either serotonin or epinephrine alone failed to induce platelet aggregation in both WT and GRK6-deficient platelets (Figure 3A). In addition, compared to the effect of GRK6 in 2-MeSADP-induced aggregation, platelet aggregation induced by co-stimulation of serotonin and epinephrine was not affected in the GRK6-deficient platelets (Figure 3B), suggesting that GRK6 was not involved in the regulation of 5HT_2A_- and α_2A_ adrenergic receptor-induced platelet aggregation.

We further evaluated the contribution of GRK6 on P2Y_1_ and P2Y_12_ receptors. Platelet aggregation induced by co-stimulation of G_q_ plus G_i_ with serotonin and 2-MeSADP in the presence of P2Y_1_-receptor antagonist MRS2179 or G_q_ plus G_z_ by 2-MeSADP in the presence of P2Y_12_-receptor antagonist AR-C69931MX and epinephrine was significantly potentiated in GRK6-deficient platelets compared to WT platelets (Figure 3C). However, the extent of the potentiation of platelet aggregation was smaller compared to the extent of platelet aggregation induced by 2-MeSADP in GRK6-deficient platelets in Figure 3B, confirming that GRK6 selectively regulates P2Y_1_- and P2Y_12_-mediated platelet functions.

### 2.3. GRK6 Plays an Important Role in ADP and PAR4 Receptor Desensitization in Platelets

**Figure 4:**
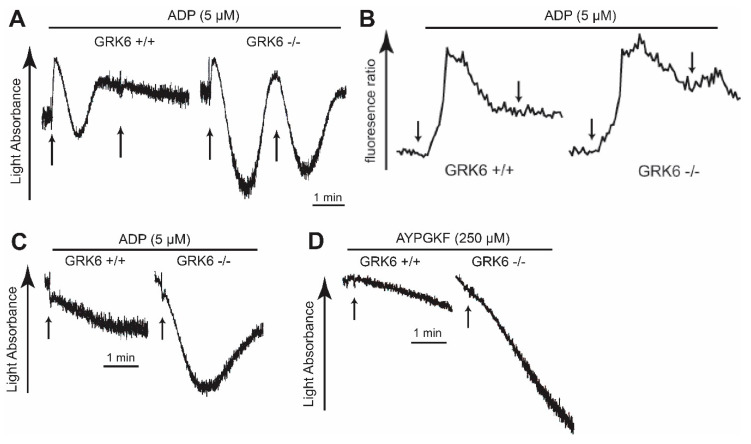
Desensitization of platelet aggregation in response to adenosine diphosphate (ADP) and AYPGKF in GRK6-deficient platelets. (**A**) Platelets from GRK6^+/+^ and GRK6^−/−^ mice were stimulated with 5 µM ADP for 3 min. Platelets were re-stimulated with 5 µM ADP for 3 min. (**B**) Fura-2-AM-loaded platelets from GRK6^+/+^ and GRK6^−/−^ mice were stimulated with 5 µM ADP for 3 min and re-stimulated with 5 µM ADP. Intracellular Ca^2+^ increase was measured. (**C**) Platelets from GRK6^+/+^ and GRK6^−/−^ mice were stimulated with 5 µM ADP after 3 min pre-incubation with 5 µM ADP. (**D**) Platelets from GRK6^+/+^ and GRK6^−/−^ mice were stimulated with 250 µM AYPGKF after 30 min pre-incubation with 250 µM AYPGKF. Results are representative of three independent experiments.

In order to determine the role of GRK6 in GPCR desensitization in platelets, we measured platelet aggregation in response to re-stimulation in the WT and the GRK6-deficient platelets. As shown in Figure 4A, re-stimulation with ADP immediately after disaggregation, 3 min following the first addition of ADP, did not induce shape change or aggregation in the WT platelets. In contrast, platelet aggregation in response to the second challenge of ADP was restored in the GRK6-deficient platelets. Moreover, there was an increase in ADP-induced Ca^2+^ release in GRK6^−/−^ platelets compared to the WT (Figure 4B). Slight increase in Ca^2+^ release was also observed in response to re-stimulation of ADP in GRK6^−/−^ platelets, indicating that GRK6 regulated ADP-induced P2Y_1_ receptor desensitization in platelets. Similarly, platelet aggregation was restored in response to ADP after pretreatment with ADP in the GRK6-deficient platelets compared to that in the WT platelets (Figure 4C), indicating that GRK6 played a role in the P2Y_1_ and P2Y_12_ receptor desensitization in platelets. In addition, platelet aggregation was restored in response to a second challenge of AYPGKF after pretreatment with AYPGKF in the GRK6-deficient platelets, whereas re-stimulation of AYPGKF failed to induce aggregation in the WT platelets (Figure 4D), suggesting that GRK6 contributed to PAR4 desensitization in platelets.

### 2.4. GPCR-Mediated Signaling Events are Potentiated in GRK6-Deficient Platelets

**Figure 5:**
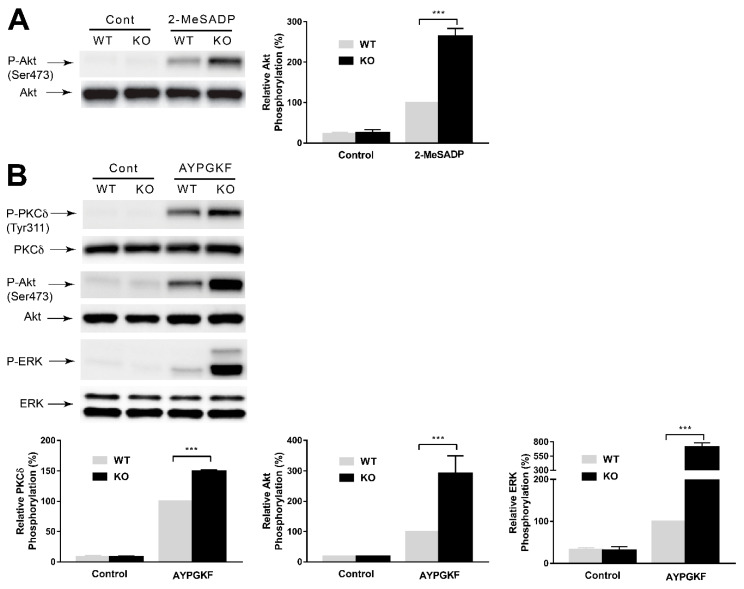
Potentiation of GPCR-mediated signaling events in GRK6-deficient platelets. Washed platelets from GRK6^−/−^ (KO) mice and GRK6^+/+^ (WT) littermates were stimulated with (**A**) 50 nM 2-MeSADP and (**B**) 100 µM AYPGKF for 2 min and probed with anti-phospho-PKCδ (Tyr^311^), anti-phospho-Akt (Ser^473^), anti-phospho-extracellular signal-related kinase (ERK), anti-Akt, anti-protein kinase Cδ (PKCδ), or anti-ERK antibodies by Western blotting. Blots are representative of three independent experiments and are presented as mean ± SE. ***, *p* < 0.005.

In order to evaluate the effect of GRK6 on platelet signaling, we stimulated platelets with 2-MeSADP and AYPGKF in the WT and the GRK6-deficient platelets and measured the phosphorylation of protein kinase Cδ (PKCδ), Akt, and extracellular signal-related kinase (ERK) as per a method described previously [22]. These proteins were selected for their known mechanism and effect on platelet activation [23,24,25,26,27]. Moreover, 2-MeSADP-induced Akt phosphorylation (Figure 5A) and AYPGKF-induced Akt, ERK, and PKCδ phosphorylations (Figure 5B) were potentiated in the GRK6-deficient platelets compared to WT platelets, suggesting a role of GRK6 in regulating both G_q_- and G_i_-mediated signaling events in platelets.

### 2.5. Contribution of GRK6 in the Platelet Function In Vivo

**Figure 6:**
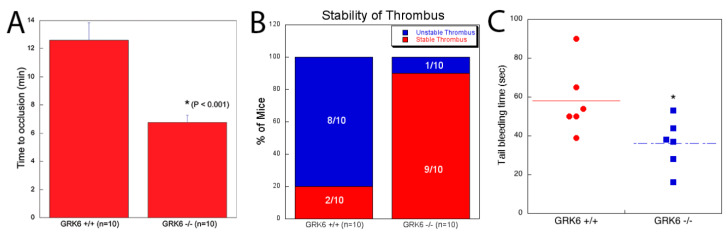
Enhanced in vivo platelet functional responses, thrombus growth, and stability in GRK6^−/−^ mice in the FeCl_3_ injury model. The carotid arteries of WT and GRK6^−/−^ mice were injured by 5% FeCl_3_ for 90 s. Flow rates through the carotid artery were measured for 30 min with a Doppler flow probe following vascular injury. (**A**) Time to occlusion of the carotid artery in GRK6^+/+^ and GRK6^−/−^ mice was measured. Data are presented as mean ± SE. Statistical analysis was performed using a Student *t* test (* *p* < 0.001). (**B**) Thrombus stability was assessed by determining the percentage of mice that maintain stable thrombi for at least 10 min. (**C**) Tail bleeding time was measured in GRK6^+/+^ and GRK6^−/−^ mice following excision of the distal 4 mm of the tail. Data are mean ± SE. *, *p* < 0.05.

ADP, thromboxane, and thrombin are important agonists that contribute to thrombus growth and stability. To evaluate the contribution of GRK6 to the platelet function in vivo, we used in-vivo thrombosis models using FeCl_3_ injury of the carotid artery [28,29] in WT and GRK6^−/−^ mice. As shown in Figure 6A, WT mice formed a thrombus 12.5 min after the vascular injury whereas the GRK6^−/−^ mice formed a thrombus 7 min after the injury, suggesting the enhanced thrombus formation in GRK6^−/−^ mice after the vascular injury. In addition, 90% of the GRK6^−/−^ mice showed stable thrombus formation, while 80% of the WT mice showed unstable thrombus formation (Figure 6B). We also measured tail-bleeding time in GRK6^+/+^ and GRK6^−/−^ mice, which reflects primary hemostatic function. We observed that average time taken for complete blockade of tail bleeding was 36 s in GRK6^−/−^ compared to 58 s in GRK6^+/+^ mice, suggesting that GRK6 negatively regulates hemostatic function in vivo (Figure 6C). Taken together, these results indicate the critical role of GRK6 in platelet function in vivo.

## 3. Discussion

GRKs are ubiquitously expressed in tissues, and most of them show activity towards a wide variety of agonist-occupied receptors in vitro. Although GRKs were known to be involved in the desensitization of GPCRs in other cell types, the functional role of these signaling molecules and their mechanisms in regulating platelet physiological events have not been fully understood. It has been demonstrated that human platelets can become refractory to activation after a major surgery, possibly leading to an increased risk of post-surgical bleeding [30], suggesting that desensitization mechanisms play a critical role in regulating platelet responsiveness. The absence of a specific inhibitor of GRK6 has made it difficult to precisely determine its role in platelet function. Therefore, we used the GRK6-deficient platelets to evaluate the role of GRK6 in platelet function and characterized the novel mechanisms involved in GPCR desensitization in platelets.

It has been shown in other cells that different GRKs are involved in the regulation of specific GPCR desensitization. It has been demonstrated that the PAR1 and PAR4 receptors are internalized after desensitization following activation in platelets, but the basis of GRKs in regulation of platelet function has not been determined [31,32]. Thus, we first have investigated whether GRK6 had a role in the regulation of GPCR-mediated platelet function. We found that platelet aggregation and dense granule secretion induced by GPCR agonists including 2-MeSADP, U46619, AYPGKF, and thrombin were potentiated in the GRK6-deficient platelets compared to those in the WT platelets. We also observed that the GPCR-mediated platelet response was potentiated in the aspirin-treated GRK6-deficient platelets, indicating that the enhanced platelet aggregation in the GRK6-deficient platelet is not due to the effect of GRK6 on the positive feedback effect of generated TxA_2_ (data not shown). Consistently, both JON/A binding and P-selectin expression that recognizes integrin αIIbβ3 activation and α-granule secretion, respectively, were significantly enhanced upon stimulation with AYPGKF in GRK6-deficient platelets. However, GPVI agonist CRP-induced platelet aggregation and secretion were not affected in the GRK6-deficient platelets suggesting that GRK6 did not have any role in non-GPCR-mediated platelet function. Taken together, our data suggest that GRK6 plays an important role in the regulation of GPCR-mediated platelet function.

GRK6 had the least effect on the TxA_2_-induced platelet aggregation and secretion. TxA_2_ receptor exists as two alternatively spliced variants: TPα (platelets) and TPβ (endothelial cells), that differ in length and sequence of the C-terminal tail [33]. Different GRK isoforms have been shown to have different affinities toward these variants in other cells [34]. In contrast, it has been reported that the C-terminus of TPα is not capable of being phosphorylated by GRKs in other nucleated cell types [19,20]. Differences in tail sequence and number of the phosphorylation-amenable serine residues also allow for differential affinity for its agonists. It is known that TPα has a shorter C-terminus with lesser phosphorylatable serine residues, which might lead to decreased affinity towards TPα receptor than other GPCRs in platelets. Another possibility may be that receptors tend to phosphorylate and internalize rapidly following stimulation with U46619, which does not allow sufficient binding of GRK6 for phosphorylation of the residue [35]. However, further study is needed to identify the relation between serine residues and affinity, responsible for the functional regulation of GPCRs by GRK6 in platelets.

It has been shown that co-stimulation of the G_q_-coupled P2Y_1_ and the G_i_-coupled P2Y_12_ receptors is essential for ADP-induced platelet aggregation [1,36]. Moreover, co-stimulation of serotonin and epinephrine induces aggregation in platelets. The α_2A_ adrenergic receptor, when stimulated with epinephrine, couples primarily to G_z,_ which belongs to the G_i_ class of the G proteins [37,38] that can mimic the P2Y_12_ receptor activation pathway. On the other hand, serotonin acts on the 5HT_2A_ receptor, which bypasses the P2Y_1_ receptor to supplement G_q_ signaling [39]. Although concomitant signaling through the serotonin and epinephrine causes platelet aggregation, neither of them can cause platelet aggregation by itself [1,40,41]. Interestingly, serotonin only induced shape change while epinephrine induced neither shape change nor aggregation in both GRK6-deficient and WT platelets. Moreover, we found that in contrast to an ADP-induced aggregation, platelet aggregation induced by co-stimulation of serotonin and epinephrine was not affected in the GRK6-deficient platelets. These suggest that GRK6 is not involved in the regulation of α_2A_ and 5HT_2A_ receptors and plays a role in regulating specific GPCR function.

It is possible that the enhanced platelet response in the GRK6-deficient platelets is due to an altered expression of receptors in the GRK6-deficient platelets. To clarify this, we assessed the PAR4 receptor expression in both the WT and the GRK6-deficient platelets and found that the PAR4 receptor level in GRK6-deficient platelets was similar to that in the WT platelets. This confirms that the expression of PAR4 receptor is not affected by the absence of GRK6.

GRK6 has been implicated in various receptor desensitizations in vivo and in vitro [42,43]. It has been suggested that re-sensitization of the platelet aggregation response to ADP is due to desensitization of the P2Y_1_ receptor, while the P2Y_12_ receptor remains functional [44]. Another study suggested that both the P2Y_1_ and P2Y_12_ receptors undergo desensitization by different mechanisms involving PKC and GRK2 and GRK6, respectively [17]. We found that re-stimulation of platelets with ADP and AYPGKF restored platelet aggregation in GRK6-deficient platelets, suggesting that GRK6 played a role in the desensitization of ADP and PAR4 receptors. At this stage, it is not clear whether the effect of GRK6 on ADP receptors is due to the desensitization of both the P2Y_1_ and P2Y_12_ receptors or the selective desensitization of either the P2Y_1_ receptor or the P2Y_12_ receptor, because ADP requires co-activation of both the P2Y_1_ and P2Y_12_ receptors. We found that platelet aggregation was potentiated in response to serotonin or epinephrine in combination with selective activation of P2Y_1_ or P2Y_12_, respectively, in GRK6-deficient mice, while serotonin and epinephrine failed to restore aggregation. We also found the increased Ca^2+^ release in response to both initial and re-stimulation of platelets with ADP in GRK6-deficient platelets compared to the WT platelets, indicating the role of GRK6 in P2Y_1_ receptor desensitization. These data confirm that the desensitization of both P2Y_1_ and P2Y_12_ receptors is responsible for platelet desensitization to ADP, and GRK6 is involved in regulation of both the P2Y_1_ and P2Y_12_ receptors in platelets.

GPCR-mediated activation of Akt, ERK, and PKCδ is important and necessary to enhance and promote platelet aggregation. Previous studies have shown that ADP-induced Akt activation is dependent on G_i_-mediated P2Y_12_ receptor stimulation [45] and AYPGKF-mediated Akt and ERK phosphorylation has been shown to be regulated by the G_i_ pathway by the secreted ADP in platelets [26]. It has been also shown that PKCδ, downstream of G_q_ pathway, plays a positive role in AYPGKF-induced platelet aggregation and dense granule secretion [25]. We found that the deletion of GRK6 potentiated ADP-induced Akt phosphorylation and AYPGKF-induced Akt, ERK, and PKCδ phosphorylation, suggesting that GRK6 regulates both the G_q_- and G_i_-mediated signaling events in platelets.

ADP, thromboxane, and thrombin are important agonists that contribute to thrombus growth and stability. Since we observed the potentiation of these agonist-induced platelet responses in GRK6-deficient platelets, we then evaluated the contribution of GRK6 to the function of platelets in vivo. We found that enhanced platelet function in GRK6^−/−^ mice resulted in stable thrombus formation and rapid time to occlusion in in vivo thrombosis models using FeCl_3_ injury of the carotid artery, indicating that GRK6^−/−^ mice are more susceptible to thrombosis. The bleeding time was also significantly reduced in GRK6^−/−^ mice. These results confirm the critical role of GRK6 in platelet function in vivo. Since GRK6 is ubiquitously expressed, we cannot rule out the contribution of GRK6 of other cells including leukocytes, endothelial cells, and smooth muscle cells on thrombus formation.

While we were preparing this manuscript, Chen et al. [46] has published a study suggesting that GRK6 regulates the hemostatic response to injury through its rate-limiting effects on GPCR signaling in platelets. Similar to our study, they have shown that GRK6 regulates P2Y_12_ and PAR4 receptor signaling. Importantly, we have investigated the effect of GRK6 on 5HT_2A_ and α_2A_ adrenergic receptor in GRK6^−/−^ platelets with serotonin and epinephrine and have found that these receptors are not regulated by GRK6 in platelets. This approach with the addition of either P2Y_1_ and P2Y_12_ receptor antagonists also helped us to confirm that both P2Y_1_ and P2Y_12_ receptors are regulated by GRK6 in platelets. Moreover, we have demonstrated the effect of GRK6 on desensitization of platelet aggregation in response to ADP and AYPGKF.

In conclusion, we demonstrate that GRK6 plays an important role in the regulation of GPCR-mediated platelet activation and in vivo thrombus formation by selectively regulating desensitization of specific GPCRs.

## 4. Materials and Methods

### 4.1. Materials

U46619, 2-MeSADP, ADP, thrombin, serotonin, epinephrine, apyrase (type V), prostaglandin E_1_ (PGE_1_), sodium citrate, and acetylsalicylic acid were from Sigma (St. Louis, MO, USA). CRP was obtained from Dr. Richard Farndale at the University of Cambridge. Phycoerythrin-conjugated antibody JON/A and FITC-conjugated anti-P-selectin antibody were from Emfret Analytics (Sterntalerweg, Wurzburg, Germany). Hexapeptide AYPGKF was custom synthesized by Invitrogen (Carlsbad, CA, USA). Fura-2-AM was from Millipore (Temecula, CA, USA). Anti-phospho-Akt (Ser^473^), anti-phospho-ERK (Thr202/Tyr204), anti-phospho-PKCδ (Tyr311), anti-Akt, anti-PKCδ, and anti-ERK antibodies were purchased from Cell Signaling Technology (Beverly, MA, USA). Horseradish peroxidase-labeled secondary antibody was bought from Santa Cruz Biotechnology (Santa Cruz, CA, USA). All other reagents were of analytical grade, and deionized water was used throughout the study.

### 4.2. Animals

All animal experiments were performed in accordance with the approval obtained from the Chungbuk National University Animal Ethics Committee (CBNUA-873-15-02, approved on July 1, 2019). GRK6^−/−^ mice were obtained from Dr. Walter Koch (Temple University, Philadelphia, PA, USA). WT littermates were used as controls. The platelet count was normal in GRK6^−/−^ mice.

### 4.3. Preparation of Murine Platelets

Blood was collected from equal number of both male and female mice and platelet was prepared as described [47]. Briefly, the collected citrated blood was centrifuged at 100× *g* for 10 min at room temperature (RT) to obtain Platelet Rich Plasma (PRP) and the obtained PRP was centrifuged at 400× *g* for 10 min to pellet the platelets. Platelet pellets were re-suspended in the Tyrode’s buffer (pH = 7.4) containing 0.05 units/mL of apyrase, and cells were adjusted to 2 × 10^8^ cells/mL.

### 4.4. Platelet Aggregation and Secretion

Agonist-induced platelet aggregation and secretion were measured using a lumi-aggregometer (Chrono-Log, Havertown, PA, USA) at 37 °C under stirring conditions (900 rpm) as per a method described previously [47]. Washed platelets (0.5 mL) were stimulated with the different agonists, and tracings of light transmission were measured. Platelet secretion was determined by measuring the release of ATP after adding the luciferin/luciferase reagent.

### 4.5. Flow Cytometry

Washed murine platelets were used to measure integrin α_II_bβ_3_ activation with phycoerythrin-conjugated JON/A antibodies and the surface exposure of P-selectin with FITC-conjugated P-selectin antibodies as per a method described previously [48]. All measurements were determined using a FACSCalibur flow cytometer (BD Biosciences, San Jose, CA, USA). Appropriate isotype controls were used.

### 4.6. Calcium Mobilization

Intracellular Ca^2+^ concentration was measured in Fura-2-AM loaded platelets as previously described [49].

### 4.7. Western Blotting

Platelets were stimulated with different agonists for the appropriate time, and phosphorylation events were measured as described previously [22]. Briefly, platelets were stimulated with 2-MeSADP or AYPGKF and the reaction was stopped by the addition of 3× sodium dodecyl sulfate (SDS) buffer. Platelet lysates were separated by 10% SDS/PAGE and transferred onto polyvinylidene difluoride (PVDF) membranes. Membranes were incubated overnight at 4 °C with anti-phospho-Akt (Ser473), anti-phospho-ERK (Thr202/Tyr204), anti-phospho-PKCδ (Tyr311), anti-Akt, anti-PKCδ, or anti-ERK antibodies. Membranes were then probed with goat anti-rabbit antibody and immune-reactivity was detected using the Fuji-Film Luminescent Image Analyzer (LAS-3000 CH, Tokyo, Japan).

### 4.8. FeCl_3_-Induced In Vivo Thrombosis Model

Adult mice (10–12 weeks old) were anesthetized and FeCl_3_-induced thrombosis was assessed as per a method described previously [29]. Briefly, the carotid artery was injured by 5% FeCl_3_ for 90 s, and blood flow through the carotid artery was measured for 30 min using the Doppler flow probe following vascular injury. The occlusion time due to thrombus formation was recorded and plotted as time to occlusion.

### 4.9. Tail Bleeding Time Assay

The tail bleeding time assay was performed as described previously [29]. Briefly, a longitudinal cut was made in anesthetized mouse’s tail and tail was immediately immersed in 0.9% isotonic saline at 37 °C. The bleeding time was defined as the time required for the blockade of blood flow.

### 4.10. Statistical Analysis

All statistical analyses were performed using the Prism software (version 3.0). Data have been presented as mean ± standard error (SE). Statistical significance was determined by the Student’s *t*-test.

## Abbreviations

GPCR	G protein-coupled receptor
GRK	G protein-coupled receptor kinase
ADP	Adenosine diphosphate
CRP	Collagen-related peptide
PAR	Protease-activated receptor
ERK	Extracellular signal-related kinase
PKCδ	Protein Kinase Cδ
TP	Thromboxane receptor
PRP	Platelet-rich plasma
